# Progression‐free survival at 3 years is a reliable surrogate for 5‐year overall survival for patients suffering from locally advanced esophageal squamous cell carcinoma

**DOI:** 10.1002/cam4.4751

**Published:** 2022-04-17

**Authors:** Yu‐Xian Yang, Yu‐Zhen Zheng, Tian‐Tian Gao, Shi‐Liang Liu, Mian Xi, Meng‐Zhong Liu, Jun‐Ye Wang, Shu‐Nan Qi, Yong Yang, Lei Zhao

**Affiliations:** ^1^ Department of Radiation Oncology, Sun Yat‐sen University Cancer Center State Key Laboratory of Oncology in South China; Collaborative Innovation Center for Cancer Medicine Guangzhou China; ^2^ Department of Thoracic Surgery, The Sixth Affiliated Hospital Sun Yat‐sen University Guangzhou China; ^3^ Department of Thoracic Surgery, Sun Yat‐sen University Cancer Center State Key Laboratory of Oncology in South China; Collaborative Innovation Center for Cancer Medicine Guangzhou China; ^4^ Department of Radiation Oncology, National Cancer Center/Cancer Hospital, Chinese Academy of Medical Sciences (CAMS) and Peking Union Medical College (PUMC) Center for Cancer Precision Medicine, CAMS and PUMC, National Institute of Biological Sciences, Collaborative Innovation Center for Cancer Medicine Beijing China; ^5^ Department of Radiation Oncology Fujian Medical University Union Hospital Fuzhou China

**Keywords:** esophageal squamous cell carcinoma, overall survival, progression‐free survival at 3 years

## Abstract

**Background:**

Despite 3‐year survival being used as a primary endpoint in some randomized controlled trials (RCTs), limited evidence supports the use of intermediate endpoints to evaluate the effect of new therapies in esophageal squamous cell cancer (ESCC). This study aimed to systematically evaluate progression‐free survival at 3 years (3‐year PFS) and overall survival (OS) among patients with ESCC.

**Methods:**

We identified 528 patients newly diagnosed with locally advanced ESCC who received definitive radiotherapy. OS was compared with an age‐ and sex‐matched general Chinese population using the standardized mortality ratio (SMR). Regression analysis was used to validate the correlation between PFS and OS using published data.

**Results:**

The annual risk of progression decreased to 11.5% after 3 years. Patients who did not achieve 3‐year PFS had a median postprogression survival (PPS) of 7.3 months, with a 5‐year OS rate of 9.6% and a SMR of 15.0 (95% confidence interval [CI], 12.9–17.5). Conversely, the SMR for patients who achieved 3‐year PFS was 0.9 (95% CI, 0.6–1.3). We observed a significant correlation between log hazard ratio (HR) (PFS) and log HR (OS) at the trial level (*r* = 0.89; 95% CI, 0.88–0.90). The strongest correlation was observed between 3‐year PFS and 5‐year OS in RCTs and retrospective studies.

**Conclusions:**

Patients exhibiting progression within 3 years experienced poor survival, whereas patients achieving 3‐year PFS had excellent outcomes. Our study supports 3‐year PFS as a reliable primary endpoint for study design and risk stratification in locally advanced ESCC.

## Graphical abstract

1. The annual progression and death hazards decreased to less than 20% over the first 3 years for patients with locally advanced esophageal squamous cell carcinoma. 2. Patients who were progression‐free within 3 years after initial treatment experienced favorable long‐term outcomes, with overall survival times not distinguishable from that of age‐ and sex‐matched Chinese populations. Conversely, patients who experienced disease progression within 3 years had very poor prognosis. 3. 3‐year progression free survival can be used as a potential endpoint for trial design and risk stratification in future studies.

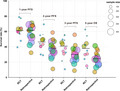

## 1 INTRODUCTION

Worldwide, approximately 3% of all cancers are diagnosed as esophageal cancer, which is the sixth most common cause of cancer‐related death globally.^1^ In China, 90% of esophageal cancer cases are esophageal squamous cell carcinoma (ESCC).^2^ Over the last two decades, the introduction of novel radiotherapy (RT) techniques and chemotherapy (CT) has resulted in major advances in treatment. Despite improvements in treatment outcomes, many patients who develop locally advanced disease suffer from relapse or progression, resulting in poor prognosis, with a median postprogression survival (PPS) of only 13 months for patients experiencing local relapse.^3^ Thus, the identification of early efficacy endpoints and new therapies in prospective trials involving patients with locally advanced ESCC are urgently required. Overall survival (OS) is an unquestionable and unbiased primary endpoint in most randomized clinical trials. However, the assessment of OS requires large sample sizes and long‐term follow‐up, and effective salvage treatment might influence the evaluation of the true effect of first‐line treatment.

OS and progression‐free survival (PFS) were identified as positively correlated in some gastrointestinal cancers.^4^, ^5^ PFS at a particular time point (such as 2 years) was found to be an important milestone to stratify patients with lymphomas and solid tumors.^6^, ^8^ However, the clinical significance of PFS at different time points in locally advanced ESCC is unknown. Moreover, the effect on OS of achieving PFS has not been studied. Herein, we aimed to examine the timing of events, posttreatment milestones, and OS among patients with locally advanced ESCC in comparison with those in the general Chinese population. Furthermore, the study validated the relationship between PFS and OS using externally published data.

## 2 PATIENTS AND METHODS

### 2.1 Individual patient selection criteria

Patients with a diagnosis of ESCC from the Sun Yat‐sen University Cancer Center between 2010 and 2017 were reviewed retrospectively. The seventh edition of the American Joint Committee on Cancer staging system was used to stage the patients. Patients with locally advanced ESCC (≥T2 or N+, M0) who had been treated with definitive RT were eligible for inclusion in the present study. The study population comprised 528 patients. The institutional review boards of the Sun Yat‐sen University Cancer Center approved the study protocol. The de‐identification of patient data meant that informed consent was not required.

### 2.2 Literature search and study selection

Studies published before January 9, 2021 were included via systematic literature searches of the Cochrane, Embase, PubMed, and Web of Science databases. The keyword was “esophageal squamous cell carcinoma AND radiotherapy,” and the search was restricted to literature published after 2000. The literature search was conducted independently by two authors (Yang YX and Zheng YZ), and the results were reviewed together with a third author (Yong Yang). The eligibility criteria included retrospective studies, phase II randomized controlled trials (RCTs), and phase III RCTs that investigated the long‐term survival of patients with locally advanced ESCC who received definitive RT or chemoradiotherapy. Studies that met any of the following criteria were excluded: patients with ESCC constituting <80% of the total sample size, nonlocally advanced ESCC, phase I trial, not receiving RT, inadequate long‐term survival data, repeated reports, non‐English studies, and retrospective studies with a sample size of <100 patients. The seven domains in the Cochrane Collaboration tool were used to assess the risk of bias in the eligible studies.^9^ All the information used in the assessment was acquired from formal publications, email contact with the trial designers, trial registry information on ClinicalTrials.gov (www.clinicaltrials.gov), and meeting abstracts. We excluded RCTs with a high risk of bias in any domain.

### 2.3 Statistical analyses

OS was defined as the period from the commencement of treatment or randomization to any cause of death. PFS was defined as the period from the commencement of treatment or randomization to the first event comprising disease failure, relapse, or any cause of death. The Epanechnikov kernel was used to smoothen the estimated hazard rates of progression and death overtime. Three‐year PFS was defined as living without progression for 3 years after treatment. PPS was defined as the period from progression to any cause of death. We also evaluated other PFS‐associated landmark time points, such as PFS at 1 and 2 years. OS was compared with sex‐ and age‐matched survival in the general Chinese population employing standardized mortality ratios (SMRs) and expected survival was estimated using a conditional approach via the survival package in R. Time to event data comparisons between two groups of patients were analyzed using Kaplan–Meier survival curves.

The relationship between OS and PFS was further validated by analyzing published data. We first obtained the treatment effects (the natural log hazard ratio [HR] of OS and PFS) and the estimates of 5‐year OS and PFS rates at 1, 2, and 3 years for each RCT arm and retrospective study using Engauge Digitizer software.^10^ We then fitted a patient size weighted linear regression (WLR) of log (HR)‐OS on log (HR)‐PFS across the RCTs. The linear association between two variables was measured using the Pearson correlation coefficient *r*. Similar analyses were performed for OS rates at 5 years on PFS rates at 1, 2, and 3 years. SPSS (version 24.0; IBM Corp., Armonk, NY, USA) and R software (version 4.04; R Foundation for Statistical Computing, Vienna, Austria) were used to perform all the statistical analyses. Statistical significance was accepted at a two‐sided *P* value of <0.05.

## 3 RESULTS

### 3.1 Patient characteristics and treatment

Table 1 lists the patients’ baseline clinical characteristics. The median age of the patients was 60 years (interquartile range [IQR], 54–67 years), with a male to female ratio of 3.7:1. The majority of the patients had good performance status (PS) and stage III (70.3%) disease. Furthermore, most patients received concurrent chemoradiotherapy (CCRT; *n* = 442, 83.7%), and only 16.3% received sequential CT and RT (*n* = 63) or RT alone (*n* = 23). The most used concurrent CT regimen was cisplatin plus paclitaxel or cisplatin plus fluorouracil (*n* = 323, 73.1%). Patients received a median radiation dose of 60 Gy (range, 60–64 Gy).

**TABLE 1:** Clinical characteristics and survival of patients with locally advanced esophageal squamous cell carcinoma treated with definitive radiotherapy

Characteristics	No. (%)	5‐year OS	1‐year PFS	2‐year PFS	3‐year PFS
		% (95% CI)	% (95% CI)	% (95% CI)	% (95% CI)
All	528	40.1 (35.7–45.1)	59.9 (55.8–64.3)	42.5 (38.4–47.1)	37.4 (33.4–42.0)
Sex					
Male	416 (78.8)	36.3 (31.4–42.0)	57.3 (52.7–62.4)	38.3 (33.8–43.5)	33.0 (28.6–38.0)
Female	112 (21.2)	53.5 (44.4–64.5)	68.3 (60.1–77.5)	56.8 (48.2–67.0)	52.6 (43.9–63.0)
Age (years)					
≤ 60	268 (50.8)	42.9 (36.5–50.4)	56.2 (50.5–62.6)	41.1 (35.4–47.6)	36.0 (30.4–42.5)
> 60	260 (49.2)	37.8 (32.0–44.8)	63.2 (57.5–69.4)	43.2 (37.4–49.8)	38.8 (33.2–45.4)
Weight loss					
< 10%	455 (86.2)	40.9 (36.1–46.3)	60.6 (56.2–65.3)	43.6 (39.1–48.5)	38.6 (34.3–43.6)
≥10%	73 (13.8)	35.8 (25.5–50.1)	54.0 (43.6–66.9)	32.9 (23.4–46.2)	26.4 (17.7–39.5)
Smoking history					
Never	201 (38.1)	44.1 (37.2–52.3)	62.7 (56.3–69.8)	46.8 (40.3–54.4)	43.3 (36.8–51.0)
Former or current	327 (61.9)	37.5 (32.0–44.0)	57.7 (52.6–63.5)	39.5 (34.4–45.3)	33.3 (28.4–39.1)
Drinking history					
Never	268 (50.8)	42.7 (36.5–49.9)	64.7 (59.2–70.8)	47.1 (41.3–53.7)	42.6 (36.9–49.3)
Former or current	260 (49.2)	37.6 (31.5–44.8)	54.5 (48.7–61.0)	37.4 (31.9–44.0)	31.8 (26.4–38.2)
ECOG PS					
0	299 (56.6)	41.3 (35.4–48.1)	62.4 (57.1–68.2)	45.5 (40.1–51.7)	40.0 (34.6–46.2)
1	221 (41.9)	40.0 (33.4–47.7)	56.4 (50.1–63.4)	38.7 (32.6–45.9)	34.5 (28.5–41.6)
2	8 (1.5)	‐	50.0 (25.0–1.0)	‐	‐
cStage (AJCC‐7th)					
Ib–II	157 (29.7)	43.7 (35.9–53.2)	67.6 (60.6–75.4)	47.7 (40.3–56.5)	43.3 (36.0–52.1)
III	371 (70.3)	38.7 (33.5–44.7)	56.3 (51.4–61.7)	40.0 (35.2–45.5)	34.6 (29.9–40.0)
Site					
Cervical	76 (14.4)	50.3 (39.6–63.8)	61.4 (51.3–73.4)	49.2 (39.0–61.9)	46.1 (36.0–59.1)
Upper	134 (25.4)	46.6 (38.1–57.1)	65.4 (57.8–74.0)	48.3 (40.4–57.9)	44.7 (36.8–54.3)
Middle	217 (41.1)	38.9 (32.3–46.8)	57.1 (50.8–64.2)	40.3 (34.1–47.6)	34.0 (28.0–41.2)
Lower	77 (14.6)	29.4 (19.9–43.6)	53.0 (42.6–65.9)	34.0 (24.5–47.1)	27.5 (18.7–40.5)
Multiple	24 (4.5)	16.3 (5.1–52.4)	56.6 (39.6–81.0)	30.5 (16.5–56.5)	21.8 (10.0–47.3)
Tumor length (cm)					
≤ 7	388 (73.5)	44.8 (39.5–50.5)	63.5 (58.8–68.5)	44.8 (40.1–50.2)	39.8 (35.1–45.2)
> 7	140 (26.5)	27.7 (20.3–37.8)	49.0 (41.3–58.2)	35.2 (27.9–44.5)	29.7 (22.7–39.0)
Initial treatment					
CCRT	442 (83.7)	43.0 (38.04–48.5)	60.1 (55.6–64.9)	44.5 (40.0–49.6)	39.1 (34.6–44.1)
SCRT	63 (11.9)	32.6 (22.40–47.36)	59.7 (48.7–73.3)	35.5 (25.4–49.7)	30.5 (20.9–44.5)
RT alone	23 (4.4)	13.5 (42.5–43.1)	47.8 (31.2–73.3)	21.7 (10.0–47.2)	21.7 (10.0–47.2)

Abbreviations: AJCC, American Joint Committee on Cancer; CCRT, concurrent chemoradiotherapy; CI, confidence interval; ECOG, Eastern Cooperative Oncology Group; OS, overall survival; PFS, progression‐free survival; PS, performance status; RT, radiotherapy; SCRT, sequential chemoradiotherapy.

### 3.2 Annual hazard rate overtime and survival

**FIGURE 1:**
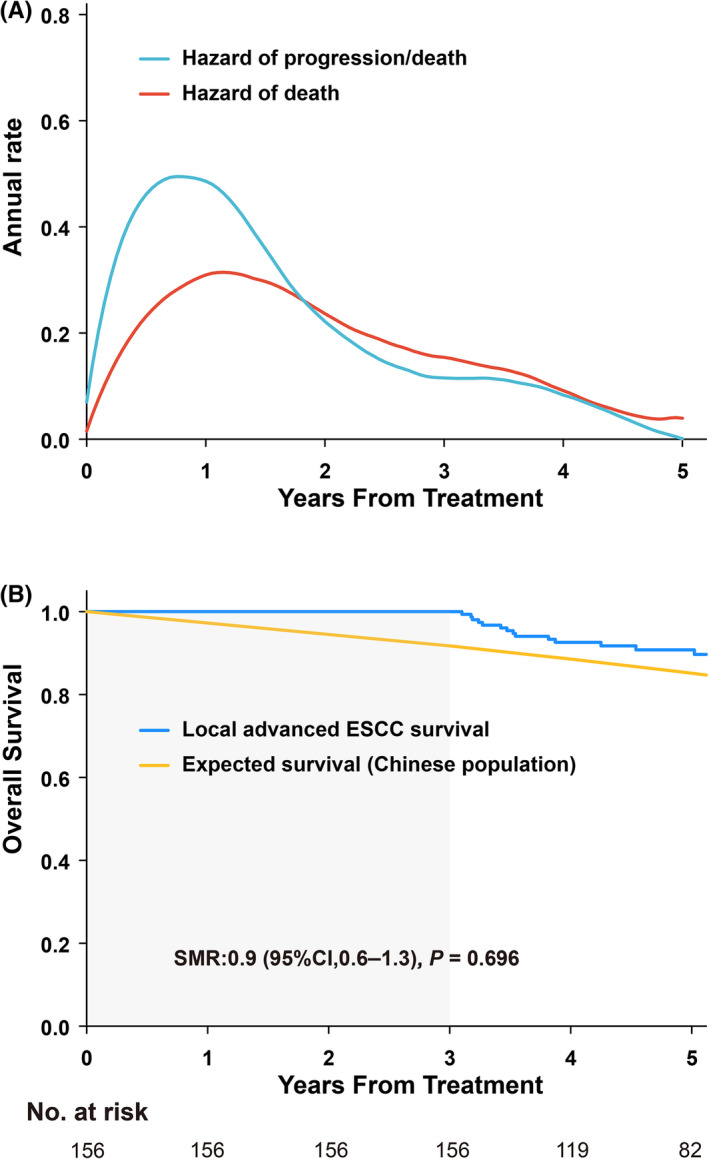
Annual hazard rate overtime and overall survival (OS) according to progression‐free survival at 3 years (3‐year PFS) in the whole cohort. (A) Annual hazard rate overtime in the whole cohort. (B) OS of patients who achieved 3‐year PFS after initial treatment vs. expected OS, based on data from sex‐ and age‐matched Chinese general population. Abbreviations: CI, confidence interval; SMR, standardized mortality ratio; ESCC, esophageal squamous cell carcinoma

After a median follow‐up of 55 months, 238 patients (45.1%) exhibited disease progression and 293 patients (55.5%) died. The estimated 5‐year OS and PFS rates were 40.1% and 31.9%, respectively. Examination of progression and risk of death showed that 92.0% of progression and 85.0% of deaths occurred within 3 years after initiation of treatment. Consistently, the smoothed hazard plot (Figure 1A) showed that the peak risk of progression and death occurred within the first 3 years. The highest annual progression (49.5%) and death (31.4%) hazards were within the first year; however, the hazards decreased to less than 20% over the first 3 years (11.5% and 15.4%, respectively). From year 4 onwards, the annual progression and death hazards decreased to less than 10%. Thus, the reliable cutoff time point for further assessment was identified as 3 years.

A total of 372 patients had sufficient follow‐up data for 3 years of assessment. Among them, 216 patients (58.1%) did not achieve 3‐year PFS, and had a PPS of 7.3 months (95% confidence interval [CI], 6.0–8.5). The 5‐year OS was only 9.6% and the SMR that compared outcomes with the expected survival in the age‐ and sex‐matched general Chinese population was 15.0 (95% CI, 12.9–17.5; *P* < 0.001). By contrast, the median OS for patients who achieved 3‐year PFS was not reached, with an observed 5‐year OS of 90.8% (Figure 1B). The SMR for patients achieving 3‐year PFS was 0.9 (95% CI, 0.6–1.3; *P* = 0.696).

**FIGURE 2:**
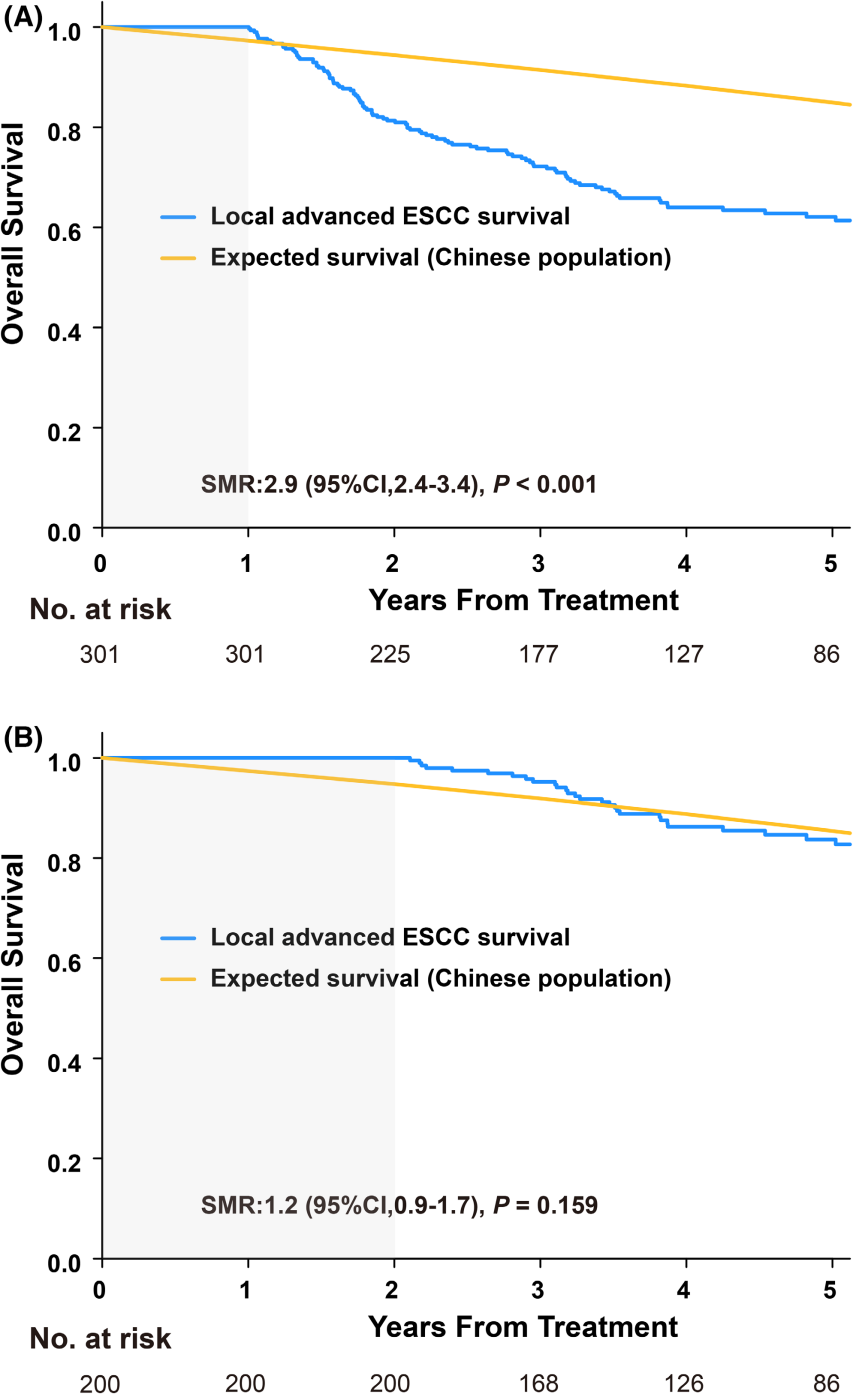
Overall survival (OS) based on progression‐free survival at 1 year (1‐year PFS) and 2 years (2‐year PFS) in the whole cohort. (A) OS of patients who achieved 1‐year PFS after initial treatment versus expected OS based on data from sex‐ and age‐matched Chinese general population. (B) OS of patients who achieved 2‐year PFS after initial treatment versus expected OS based on data from sex‐ and age‐matched Chinese general population. Abbreviations: CI, confidence interval; SMR, standardized mortality ratio; ESCC, esophageal squamous cell carcinoma

For sensitivity analysis, outcomes were examined using other landmark PFS time points: 1‐year PFS and 2‐year PFS. The 5‐year OS rate for those patients who achieved the time points continued to increase from 62.1% (1‐year PFS; Figure 2A) to 83.7% (2‐ year PFS; Figure 2B). In contrast, after progression, there was little difference in the median OS, irrespective of the chosen time point (6.4 months vs. 7.0 months).

### 3.3 External validation of the association between OS and PFS

**FIGURE 3:**
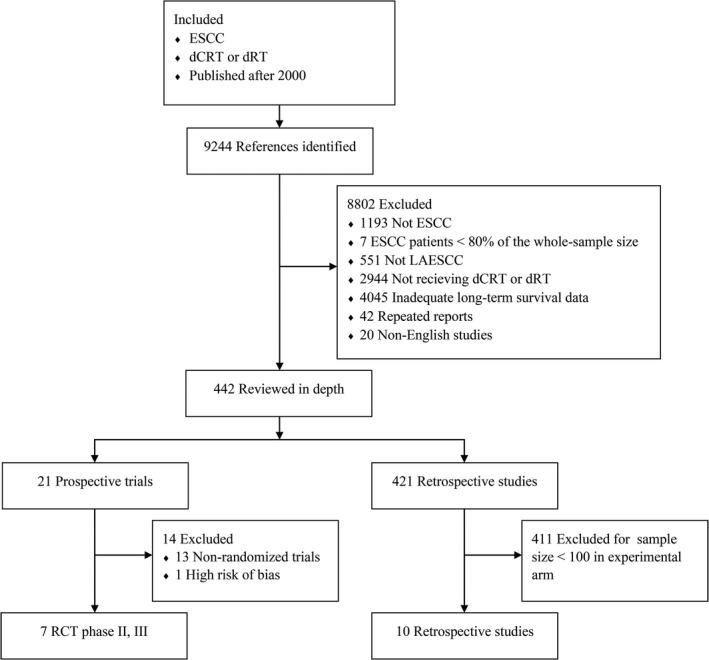
Study selection flow chart. PRISMA flow chart for phase II and III RCTs and retrospective studies. Abbreviations: RCTs, randomized controlled trials; PRISMA, preferred reporting items for systematic reviews and meta‐analyses

**FIGURE 4:**
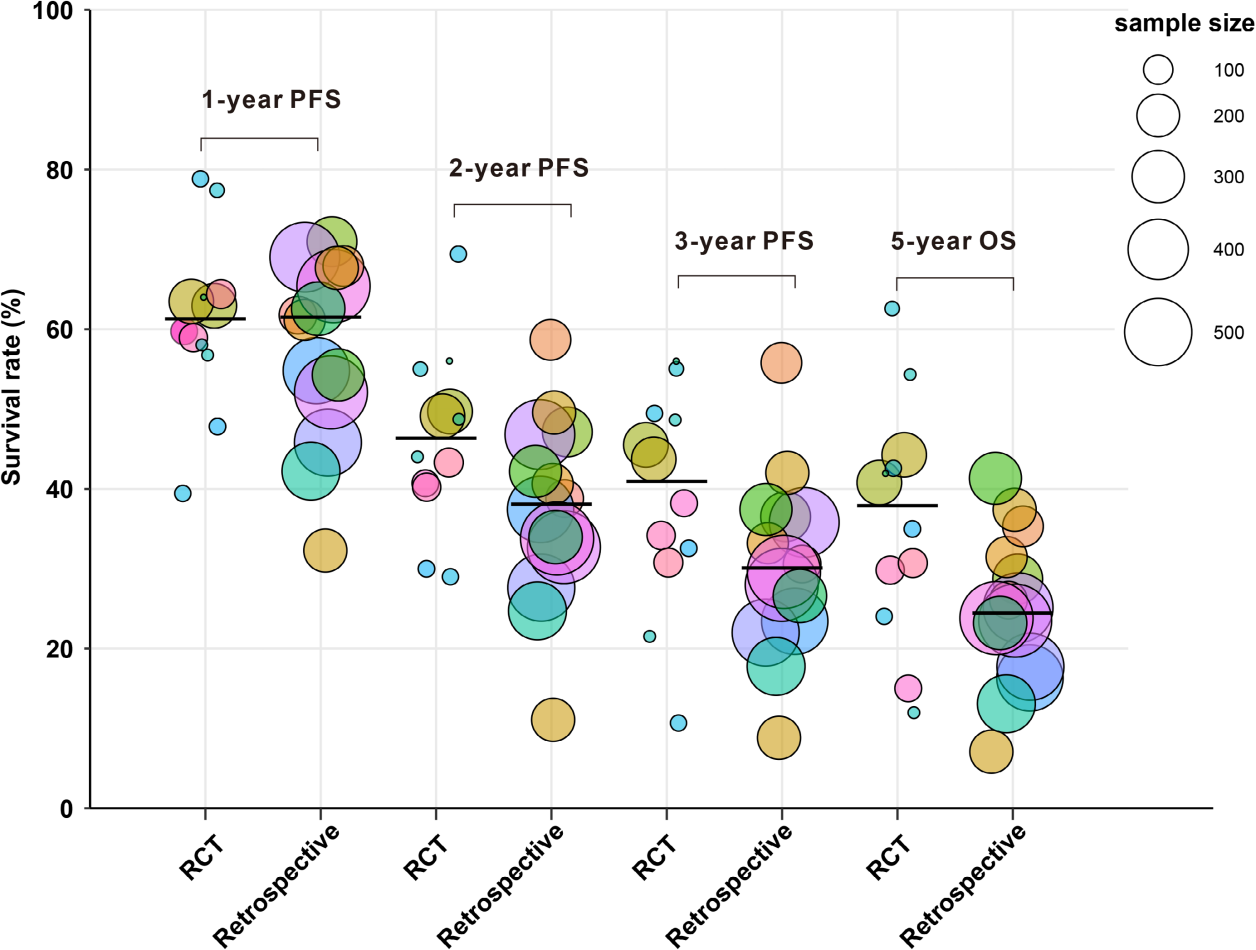
Survival rates reported in RCTs and retrospective studies. The black line represents the median survival rate. Abbreviations: OS, overall survival; PFS, progression‐free survival; RCTs, randomized controlled trials

**FIGURE 5:**
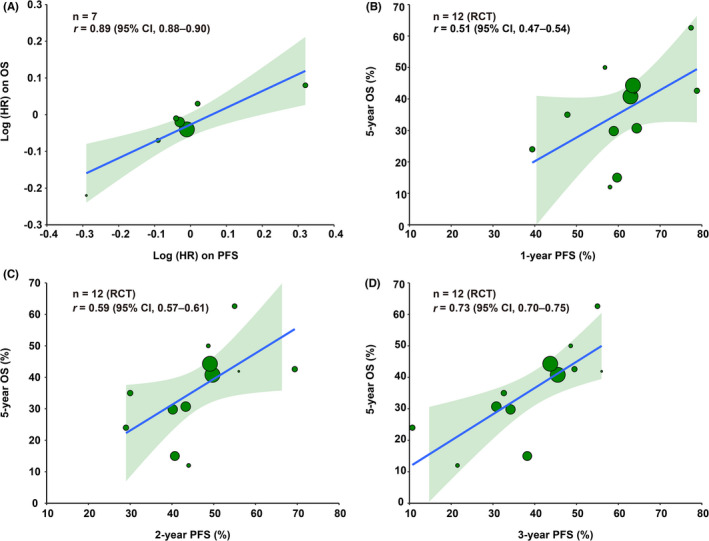
Trial‐ and arm‐level correlation between OS and PFS in RCTs. (A) Trial‐level correlations between the HR for OS and that of PFS. (B–D) The treatment arm‐level associations between 1‐, 2‐, and 3‐year PFS and 5‐year OS in RCTs. The size of the circle is proportional to the number of patients in each comparison. The fitted weighted linear regression line is shown in blue and its 95% CI is shown as a light green zone. *n* represents the number of PFS HR and OS HR pairs. *r* represents the correlation coefficient. Abbreviations: CI, confidence interval; HR, hazard ratio; RCTs, randomized controlled trials; OS, overall survival; PFS, progression‐free survival

To validate the results, the relationship between OS and PFS was analyzed using published data. A total of 9244 references were screened, and 446 were reviewed in depth (Figure 3). Twenty‐one prospective trials and 421 retrospective studies were eligible for further selection. Thirteen prospective trials were excluded because they were nonrandomized trials, and 411 retrospective studies were excluded because their sample sizes were <100. We excluded one RCT because it had a sample size far below the statistical requirements and thus had a high risk of bias (Figure S1).^11^ Finally, we included 7 RCTs^12^, ^18^ and 10 retrospective studies^19^, ^28^ for trial‐ and treatment arm‐level analyses (Tables S1 and S2). Patient survival in the RCTs was superior to that in the retrospective studies, regardless of the time point (1‐, 2‐, and 3‐year PFS and 5‐year OS). Most retrospective studies had larger sample sizes (Figure 4). At the RCT trial level, treatment effects were measured using the log HR for OS and PFS. Log HR (OS) and log HR (PFS) correlated significantly (*r* = 0.89; 95% CI, 0.88–0.90; Figure 5A). We also tested the endpoint correlations (1‐, 2‐, and 3‐year PFS and 5‐year OS) using trial‐level estimates. The *r* values from the WLR of 5‐year OS on the 1‐, 2‐, and 3‐year PFS rates across the trials and treatment arms were 0.51 (95% CI, 0.47–0.54; Figure 5B), 0.59 (95% CI, 0.57–0.61; Figure 5C), and 0.73 (95% CI, 0.70–0.75; Figure 5D), respectively.

**FIGURE 6:**
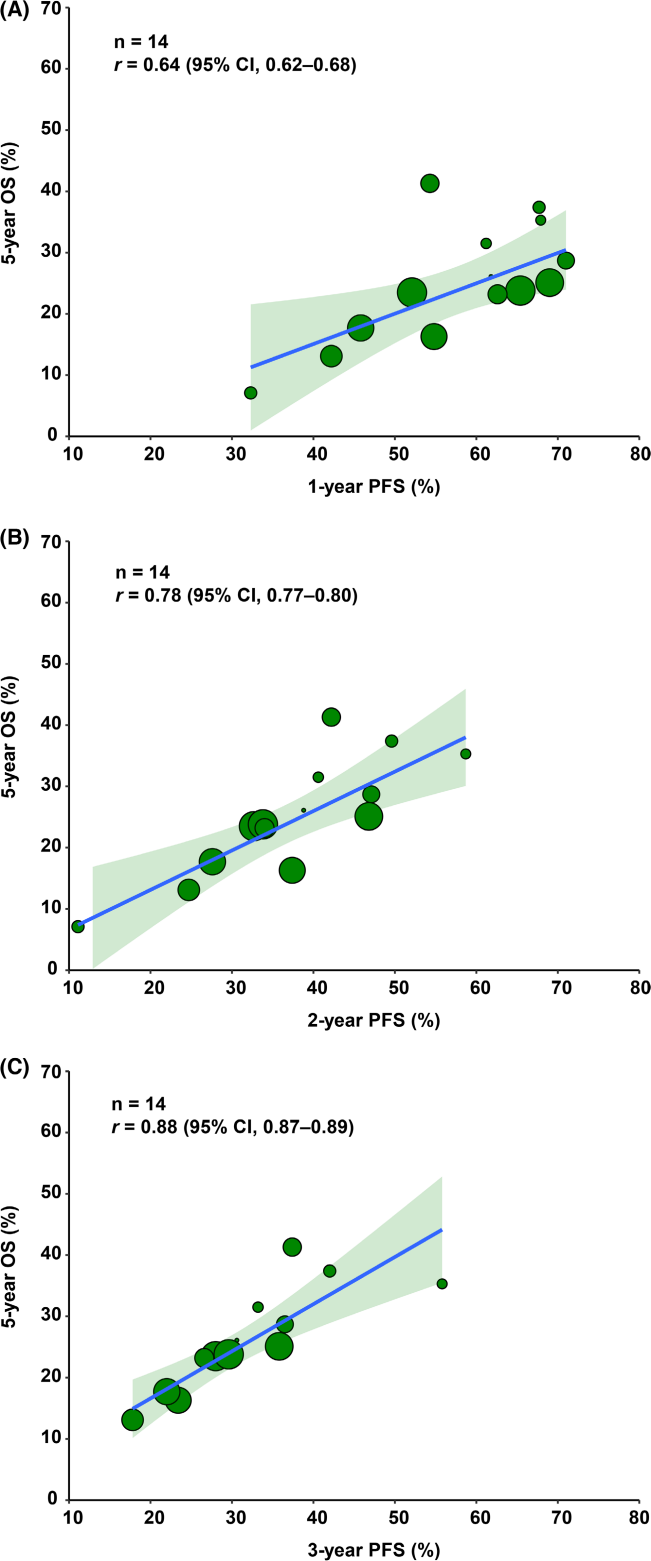
Treatment arm‐level correlation between 1‐, 2‐, and 3‐year PFS and 5‐year OS in retrospective studies. (A‐C) The treatment arm‐level associations between 1‐, 2‐, and 3‐year PFS and 5‐year OS in retrospective studies. The circle size is proportional to the number of patients in each treatment arm. The solid blue line indicates the fitted weighted linear regression line. The light green zone represents its 95% CI. *n* indicates the number of treatment arms. *r* indicates the correlation coefficient. Abbreviations: PFS, progression‐free survival; OS, overall survival; CI, confidence interval

From the retrospective studies, we used 14 treatment arms for further validation. The 1‐year (*r* = 0.64; 95% CI, 0.62–0.68; Figure 6A), 2‐year (*r* = 0.78; 95% CI, 0.77–0.80; Figure 6B), and 3‐year PFS (*r* = 0.88; 95% CI, 0.87–0.89; Figure 6C) correlated linearly with the 5‐year OS. These findings indicated that 3‐year PFS is a favorable intermediate endpoint of the 5‐year OS in locally advanced ESCC.

## 4 DISCUSSION

To the best of our knowledge, this study is the first to systematically examine PFS‐based endpoints in patients with locally advanced ESCC treated primarily with RT. Patients whose disease progressed within 3 years after the initial treatment had a substantially increased risk of death, and their PPS was very poor. Conversely, patients who were progression free for up to 3 years had a favorable 5‐year OS, with very similar OS times to those of age‐ and sex‐matched populations. The strong association between 3‐year PFS and 5‐year OS was maintained irrespective of the inclusion of RCTs or retrospective studies of patients treated with RT. Together, these results indicate that 3 years provides a clear benchmark for caregivers, patients, and clinicians to evaluate the success of initial treatment and might facilitate the design of clinical trials for locally advanced ESCC.

The introduction of CCRT and high‐precision RT techniques for locally advanced esophageal carcinoma have been the most important treatment advances.^29^ The use of paclitaxel‐based CCRT^30^, ^32^ improved survival in phase I/II trials; however, a phase III trial found that, compared with the standard regimen, the paclitaxel plus fluorouracil regimen did not significantly prolong OS.^16^ Efficacy intermediate endpoints, such as PFS and disease‐free survival (DFS), are needed to scale down the evaluation time for effective regimens and to allow ineffective strategies to be abandoned without prolonged evaluation. However, because of geographical variation and the heterogeneity of radiation doses, limited phase III trials make the analysis of formal surrogate endpoints difficult. The pivotal study by Ronellenfitsch et al. on neoadjuvant treatment of gastroesophageal adenocarcinoma showed a strong correlation between DFS and OS; however, DFS at different time points was not evaluated at the individual level.^33^ In the present study, 3 years was identified as an important endpoint because 92% of progression occurred within the first 3 years after initial therapy. Moreover, individuals who survived without progression to this time point generally experienced a normal life expectancy (5‐year OS, 90.8% and SMR, 0.9). Similarly, in the CROSS and NEOCRTEC5010 trials,^34^, ^35^ disease progression beyond 3 years after initial treatment was less than 15%.

Current approaches to improve ESCC outcomes focus on addressing key mutations and pathways involved in ESCC, for example, programmed death receptor 1/programmed cell death‐ligand 1 (PD‐1/PD‐L1) and epidermal growth factor receptor (EGFR) signaling.^36^, ^39^ These could be used for risk stratification and the identification of novel therapeutic targets. Targeting patients with poor survival, identified by a combination of both genetic and clinical factors, has become a priority for defining patient groups. Once patient groups are defined, improving outcomes with CCRT is required and feasible in a timeframe suitable for drug development. Despite the lack of high‐level evidence demonstrating an appropriate surrogate, over the past decade, some RCTs have reported 3‐year survival to be an important endpoint.^16^, ^35^ Among patients with ESCC who did not achieve 3‐year PFS, the median PPS was only 7.3 months. This indicates that further treatment salvaged a few patients successfully. In a recent study in which 64 patients suffering from ESCC experienced local relapse following definitive RT, the median PPS was only 9.5 months for patients without salvage surgery.^3^ Furthermore, previous studies confirmed that when the median PPS was short (<9 months), there was a better correlation between OS and PFS in solid cancers.^40^ Based on our results, we believe that 3‐year PFS should be further validated in RCTs as a reliable efficacy intermediate endpoint for patients with locally advanced diseases.

This study had the following limitations. First, our results were based on patients with locally advanced stage disease who were treated mainly using CCRT; therefore, extrapolation of the results to other stages or treatments would be speculative. Second, salvage treatment was not assessed after progression. This might impact the strength of the correlation between OS and PFS. Moreover, such information is not routinely collected. Last, the study design did not allow us to assess 3‐year PFS using individual patient data from RCTs. Comparison with other collaborative data will provide further insights into the utility and importance of this endpoint.

## 5 CONCLUSION

In conclusion, patients with newly diagnosed locally advanced ESCC treated with definitive RT and who were progression free at 3 years posttreatment have excellent outcomes, with an OS similar to that of the age‐ and sex‐matched Chinese general population. Our findings support the use of 3‐year PFS as a reliable primary endpoint that should be taken into account in future retrospective studies to evaluate new therapeutics and could be used for risk stratification.

## Conflict of Interest

All authors declare no competing interests.

## AUTHOR CONTRIBUTIONS

Conception and design: Lei Zhao, Yong Yang. Financial support: Yong Yang, Lei Zhao. Administrative support: Yong Yang, Lei Zhao. Provision of study material or patients: Shi‐Liang Liu, Mian Xi, Meng‐Zhong Liu, Jun‐Ye Wang, Yong Yang, and Lei Zhao. Collection and assembly of data: Yu‐Xian Yang, Yu‐Zhen Zheng, Tian‐Tian Gao, Yong Yang, and Lei Zhao. Data analysis and interpretation: Yu‐Xian Yang, Yu‐Zhen Zheng, Tian‐Tian Gao, Shu‐Nan Qi, Yong Yang, and Lei Zhao. Manuscript writing: All authors. Final approval of manuscript: All authors. Accountable for all aspects of the work: All authors.

## ETHICS STATEMENT

The study protocol was approved by the institutional review boards of the Sun Yat‐sen University Cancer Center. The requirement for informed consent was waived because of the deidentification of patient data.

## Supporting information


Figure S1

Table S1

Table S2

## Data Availability

The data that support the findings of this study are available from the corresponding author upon reasonable request.
