# Supplementary figures and images for: Progression‐free survival at 3 years is a reliable surrogate for 5‐year overall survival for patients suffering from locally advanced esophageal squamous cell carcinoma
Source: Cancer Med. 2022 Apr 17;11(20):3751–60. doi: 10.1002/cam4.4751 (PMC9582670; doi:10.1002/cam4.4751)

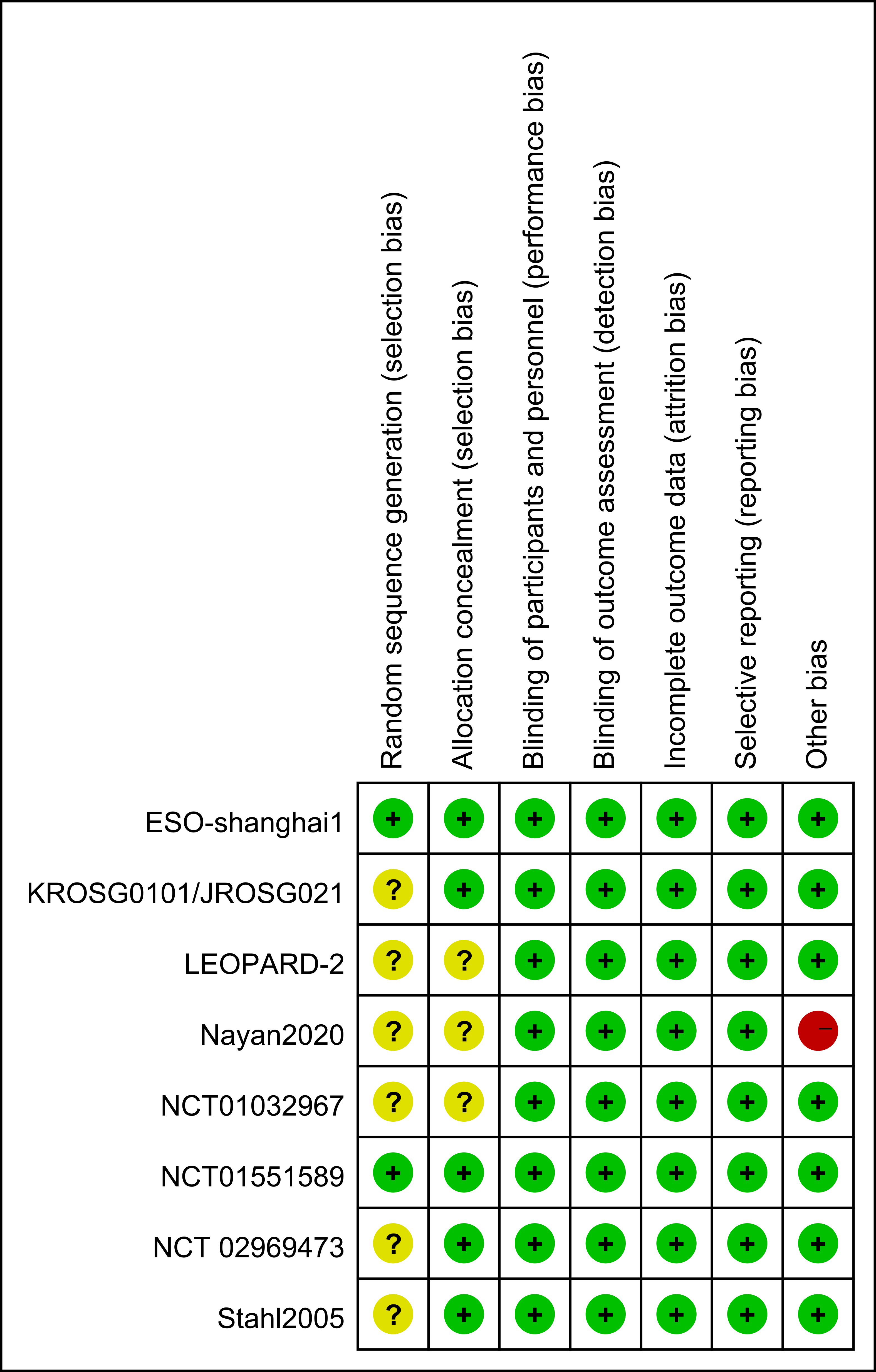

Supplement: Supplementary file 1 — Figure S1 [file CAM4-11-3751-s002.tif]
