# Supplementary material for: Progression‐free survival at 3 years is a reliable surrogate for 5‐year overall survival for patients suffering from locally advanced esophageal squamous cell carcinoma
Source: Cancer Med. 2022 Apr 17;11(20):3751–60. doi: 10.1002/cam4.4751 (PMC9582670; doi:10.1002/cam4.4751)
Supplement: Supplementary file 2 — Table S1 [file CAM4-11-3751-s003.docx]

**Supplementary Table 1.** Summary of randomized controlled trials included in trial- and treatment arm-level analyses.

| Trial | Eligibility | No. | CCRT | Concurrent regimens | PFS, % | | | | OS, % | |
| --- | --- | --- | --- | --- | --- | --- | --- | --- | --- | --- |
|  |  |  |  |  | HR | 1 - Year | 2 - Year | 3 - Year | HR | 5 - Year |
| Stahl 2005^12^ | Age < 70;  T3–4, N0–1, M0 (IUAC 1992) | 86 | Yes | EP | 2.1 | 59.7 | 40.7 | 38.2 | 1.2 | 15.0 |
| KROSG0101/JROSG021^13^ | Age < 75;  Stages II–IVA (UICC 1997) | 46 | Yes | PF | 0.91 | 47.8 | 30.0 | 32.6 | 0.98 | 35.0 |
|  |  | 45 | Yes | PF |  | 39.4 | 29.0 | 10.7 |  | 24.0 |
| NCT01032967^14^ | Age < 75;  T1–T4, N0–1, M0 | 36 | Yes | PF | 0.82 | 56.8 | 48.7 | 48.6 | 0.86 | 50.0 |
| NCT02969473^15^ | Age 18–70;  Stage II–IVA (AJCC 6th) | 45 | Yes | TP | 1.05 | 78.8 | 69.4 | 49.5 | 1.08 | 42.6 |
|  |  | 41 | Yes | PF |  | 77.4 | 55.0 | 55.0 |  | 62.6 |
| ESO-shanghai1^16^ | Age 18–75;  Stage IIA–IVB (AJCC 6th) | 219 | Yes | PF | 0.973 | 62.9 | 49.7 | 45.5 | 0.905 | 40.8 |
|  |  | 217 | Yes | TF |  | 63.5 | 49.1 | 43.7 |  | 44.3 |
| NCT01551589^17^ | Age 18–75,  Stage II–III | 94 | Yes | TP | 0.925 | 58.9 | 40.2 | 34.2 | 0.95 | 29.8 |
|  |  | 98 | Yes | TP |  | 64.4 | 43.3 | 30.8 |  | 30.7 |
| LEOPARD-2^18^ | Age 18–75;  T1–4; N0-2; M1a (AJCC 7th) | 32 | Yes | PF + cetuximab | 0.51 | 64.0 | 56.0 | 56.0 | 0.60 | 41.9 |
|  |  | 36 | Yes | PF |  | 58.0 | 44.0 | 21.5 |  | 12.0 |

Abbreviations: PS: Eastern Cooperative Oncology Group (ECOG) performance status; KPS: Karnofsky performance status; PFS: progression-free survival; HR: hazard ratio; OS: overall survival; IUAC: International Union Against Cancer; RT: radiotherapy; AJCC: American Joint Committee on Cancer; ENI: elective nodal irradiation; IFI: involved-field irradiation; UICC: Union for International Cancer Control; EP: etoposide + cisplatin; PF: cisplatin + fluorouracil; TF: taxane + fluorouracill; TP: taxane + cisplatin.
