# Supplementary material for: Progression‐free survival at 3 years is a reliable surrogate for 5‐year overall survival for patients suffering from locally advanced esophageal squamous cell carcinoma
Source: Cancer Med. 2022 Apr 17;11(20):3751–60. doi: 10.1002/cam4.4751 (PMC9582670; doi:10.1002/cam4.4751)
Supplement: Supplementary file 3 — Table S2 [file CAM4-11-3751-s001.docx]

**Supplementary Table 2.** Summary of retrospective studies included in analyses.

| Trial | Eligibility | No. | CCRT (%) | Concurrent  regimens | PFS, % | | | OS, % |
| --- | --- | --- | --- | --- | --- | --- | --- | --- |
|  |  |  |  |  | 1 - Year | 2 - Year | 3 - Year | 5 - Year |
| Zhang 2014^19^ | Stage I–IV (AJCC 6th) | 204 | Yes (100) | PF or TP | 67.7 | 49.6 | 42.0 | 37.4 |
| TAN 2015^20^ | Stage I–IV (UICC 6th) | 592 | Yes (33.8) | PF or TP | 52.1 | 32.7 | 28.0 | 23.5 |
| Zhou 2017^21^ | Age ≤ 75 years;  Stage II-IV (UICC 6th) | 313 | Yes (100) | PF | 62.6 | 34.0 | 26.6 | 23.2 |
|  |  | 204 |  |  | 32.3 | 11.1 | 8.8 | 7.1 |
| Xi 2017^22^ | Age 18­–75 years;  Stage II-III (AJCC 7th) | 182 | Yes (100) | T/P-base | 67.9 | 58.7 | 55.8 | 35.3 |
| Chen 2018^23^ | Age 18–75 years;  Stage II-III (AJCC 6th) | 272 | Yes (100) | PF | 71.0 | 47.1 | 36.5 | 28.7 |
|  |  | 540 |  |  | 69.0 | 46.8 | 35.8 | 25.1 |
| Chen 2018^24^ | T1-4; N1-3; M0 ( AJCC 7th) | 369 | Yes (100) | PF | 42.2 | 24.7 | 17.8 | 13.1 |
| Xu 2018^25^ | Stage I–IV (AJCC 8th ) | 596 | Yes (64.4) | PF or TP | 65.4 | 33.8 | 29.6 | 23.8 |
| Li 2019^26^ | Stage I–IVA (AJCC 7th) | 157 | N/A | - | 61.8 | 38.8 | 30.6 | 26.1 |
|  |  | 487 |  |  | 54.8 | 37.4 | 23.4 | 16.3 |
| Luo 2019^27^ | Stage I–IV (AJCC 6th) | 497 | Yes (57.1) | PF or TP | 45.8 | 27.6 | 22.0 | 17.7 |
|  |  | 186 |  |  | 61.2 | 40.6 | 33.2 | 31.5 |
| Lan 2020^28^ | Stage I–IVA (AJCC 8th) | 297 | Yes (100) | T/P-base | 54.3 | 42.2 | 37.4 | 41.3 |

Abbreviations: PS: Eastern Cooperative Oncology Group (ECOG) performance status; KPS: Karnofsky performance status; PFS: progression-free survival; HR: hazard ratio; 1-Y: 1-year; 2-Y: 2-year; 3-Y: 3-year; OS: overall survival. AJCC: American Joint Committee on Cancer; RT: radiotherapy; ENI: elective nodal irradiation; IFI: involved-field irradiation; UICC: Union for International Cancer Control; NLR: neutrophil-to-lymphocyte ratio. PF: platinum + fluorouracil; TF: taxane + fluorouracill; TP: taxane + platinum; T-base: taxane-base; P-base: platinum-base.
